# Engaging youth in research planning, design and execution: Practical recommendations for researchers

**DOI:** 10.1111/hex.12795

**Published:** 2018-06-01

**Authors:** Lisa D. Hawke, Jacqueline Relihan, Joshua Miller, Emma McCann, Jessica Rong, Karleigh Darnay, Samantha Docherty, Gloria Chaim, Joanna L. Henderson

**Affiliations:** ^1^ Centre for Addiction and Mental Health Toronto ON Canada; ^2^ University of Toronto Department of Psychiatry Toronto ON Canada

**Keywords:** mental health, patient‐centred research, research methodology, youth engagement

## Abstract

**Context:**

Engaging youth as partners in academic research projects offers many benefits for the youth and the research team. However, it is not always clear to researchers how to engage youth effectively to optimize the experience and maximize the impact.

**Objective:**

This article provides practical recommendations to help researchers engage youth in meaningful ways in academic research, from initial planning to project completion. These general recommendations can be applied to all types of research methodologies, from community action‐based research to highly technical designs.

**Results:**

Youth can and do provide valuable input into academic research projects when their contributions are authentically valued, their roles are clearly defined, communication is clear, and their needs are taken into account. Researchers should be aware of the risk of tokenizing the youth they engage and work proactively to take their feedback into account in a genuine way. Some adaptations to regular research procedures are recommended to improve the success of the youth engagement initiative.

**Conclusions:**

By following these guidelines, academic researchers can make youth engagement a key tenet of their youth‐oriented research initiatives, increasing the feasibility, youth‐friendliness and ecological validity of their work and ultimately improve the value and impact of the results their research produces.

## 1 INTRODUCTION

There are increasing calls for engaging youth in the development and execution of mental health services for them,^1^ but also the research being conducted about them.^2^, ^3^ While traditionally youth have been primarily research participants, youth engagement practices call for them to be brought into the research process, as full research partners when possible.^3^ Youth can be engaged in research in a variety of ways, reflecting their level of interest, availability, commitment and skill.^4^, ^5^ Youth‐adult partnerships in research provide many reciprocal benefits, such as skill development, empowerment and social engagement for the youth, as well as an increase in the feasibility, youth‐friendliness and ecological validity of the research.^3^, ^4^, ^5^ Youth engagement in research can be accomplished through a variety of approaches, such as community‐based participatory research designs and youth‐led research designs in which youth are directly conducting research about youth.^6^, ^7^, ^8^, ^9^ However, youth can also be brought into traditional academic research approaches and projects led by established academic researchers, requiring the researchers to adjust their practices to maximize the youth engagement experience.

At the Margaret and Wallace McCain Centre for Child, Youth and Family Mental Health at the Centre for Addiction and Mental Health, youth engagement in research is a key tenet to operations. This has led to the development of the McCain Model of Youth Engagement^22^, which provides for a variety of levels of youth engagement; the model includes high engagement for a small number of youth, moderate engagement for a moderate number of youth and more limited, short‐term engagement for a large number of youth. For a detailed discussion of the McCain Model of Youth Engagement, see Heffernan et al., 2017. Applying the McCain Model, youth are engaged in a wide variety of research projects in the McCain Centre. By engaging youth in academic research, the research about them has become more attuned to their needs and more feasible to conduct, setting the stage for success.

Despite a growing interest in youth engagement in research, it has not always been clear to researchers how to best engage youth in research work. Some may struggle to engage youth in meaningful ways. For example, they may not know how to adapt their procedures to give youth meaningful opportunities to express their views and to best use youth feedback to inform their research. While they may look to the emerging literature on patient engagement^10^, ^11^ and youth engagement in research^2^, ^12^ to understand the importance and value of youth engagement, the literature falls short in providing practical guidelines on the best way to engage young people in youth‐friendly ways in complex research projects.

### 1.1 Objective

This article takes a practical look at youth engagement practices in academic research endeavours. Targeting researchers, we draw on our experience, ongoing discussions with the youth members of our team (including co‐authors Relihan, Miller, McCann, and Rong), consultation with our standing Youth Advisory Group, and the literature on youth engagement and youth‐adult partnerships to offer suggestions to help researchers engage youth in meaningful ways to inform the planning, design and execution of research projects. Separate from youth‐led and participatory research approaches, in which youth are guided in leading their own research, these suggestions focus on integrating youth into teams of experienced academic researchers to draw on the reciprocal benefits for both youth and academics.

## 2 HOW TO ENGAGE YOUTH IN MENTAL HEALTH RESEARCH

### 2.1 The “dos”

The main tenets of youth engagement in the McCain Model of Youth Engagement^22^, as informed by the literature, are transparency, reciprocity and colearning, flexibility and recognition that it is a continually evolving process, active efforts to ensure a youth‐friendly environment and support by adults as resources/mentors. The McCain Centre experience, combined with the literature, has enabled us to identify several key elements, as outlined below, to be followed to maximize the value and feasibility of ongoing youth engagement in research environments. These steps can be followed throughout the research project when engaging youth, from the initial stages of project planning and grant application development, through to project completion, interpretation and knowledge translation activities disseminating the findings.

#### 2.1.1 Authentically value youth expertise

Youth are experts in their own experience and the realities of being a young person.^1^, ^13^ This expertise must be authentically valued in the research process.^14^ Authentic youth feedback can make many contributions to a research project, notably ensuring a youth‐friendly approach to a study, feasibility of recruitment and data collection, the meaningfulness of the results and ultimately project success as a whole. It is important for researchers to be mindful of the youth’s diversity of expertise and remember that they do not know everything about the youth they are engaging or the experiences these youth have had; it is therefore important to respectfully listen to what the youth have to say and ask questions in a non‐judgemental manner to ensure they have understood what is being conveyed. If the researcher does not authentically value the expertise of the youth and the reciprocal learning opportunities youth engagement provides,^14^ the youth may feel patronized or tokenized, and this will undermine the entire youth engagement process, limiting the research learning opportunities for both the youth and the project. Notably, tokenism occurs when youth are invited to the table, but are not truly invited into the discussion or given the opportunity to express their views and have them heard, or when their feedback is not taken into account in the decision‐making process.^15^, ^16^, ^17^

#### 2.1.2 Recognize diversity among youth

It is important to remember that youth status only reflects a general age grouping; among youth, there is of course a great deal of diversity to be found, including developmental stage, socioeconomic status, gender and sexuality, ethnic origin, minority status, culture, life‐experiences, abilities and disabilities, constraints and so much more. No single youth can represent all youth perspectives. Research projects can benefit from a rich array of voices when multiple youth are brought into the discussions, and the youth at the table reflect the diversity of society.^18^ Researchers are encouraged to remember that different youth may have varying viewpoints; while it may be impossible to take equal action in the face of conflicting views, all voices deserve to be heard, respected and considered during the youth engagement process. It is also important to be sure to engage youth from the specific demographic(s) that the research project is primarily targeting to ensure meaningful youth input.^19^

#### 2.1.3 Formally recognize contributions

One way to authentically value youth expertise is to recognize and acknowledge their contributions formally. Some examples of meaningful recognition include providing wages, an honorarium, references for job or school applications and/or certificates as appropriate.^20^ Under the McCain Model of Youth Engagement^22^, the youth who are highly engaged in McCain Centre projects on an ongoing basis receive a wage as regular staff, while those who are engaged less intensely or less frequently receive an honorarium for their services, and all are offered the opportunity to receive references and letters of support. Youth can also be offered co‐authorship on the documents to which they contribute^22^ (eg co‐authors Heffernan and Herzog of cited articles, co‐authors Relihan, Miller, McCann, and Rong of this article), which may be a meaningful form of recognition for some youth. By formally recognizing their contributions, researchers legitimize their contribution, strengthen engagement and help position youth as full participating members of the project team. It is important to consult young people in deciding how best to formally recognize their contributions in ways that are meaningful to them.

#### 2.1.4 Create meaningful opportunities and active participation

The youth should be included as active team members who authentically contribute to the overall goals of the project and decisions made,^14^ while being provided with opportunities to showcase their strengths. Valuable contributions can be gained by asking youth to participate in a genuine way that is meaningful to them.^16^ As different youth may derive meaning from different activities, it is important to discuss the youth’s goals to align their contributions with their objectives and perceptions of meaningfulness. This process requires flexibility with regard to roles; youth must be given opportunities to showcase their strengths. Examples of youth participation in research can include consulting on study materials, interview guides and scripts; assisting with study recruitment; and leading focus groups or training research staff who may be working with youth participants. Consider validating the role and impact of youth team members by asking them to copresent on the project with other members of the research team.

#### 2.1.5 Clearly define roles

As there are many opportunities for youth to be engaged in different roles in research, it is important to effectively prepare youth and adults for these roles.^4^, ^5^ Parties should share a clear understanding of the roles and responsibilities of the youth on the team, as well as the role of the researchers and other adult allies on the team. This prevents unclear expectations from developing among the youth and adult team members and sets the stage to engage youth authentically, avoiding the risk of misinterpreting the youth’s role and inappropriately calling on them to contribute in a manner in which they may not be prepared to do or may not want to do, which can lead to a feeling of tokenism and disempowerment. It is important for researchers to be open to different possibilities for youth participation and to take youth seriously in their role as an expert on the project.^4^, ^5^ Defining roles is important not only when bringing youth onto projects, but also when bringing new team members (youth or adults) into the team or when inviting special guests to meetings. For example, roles may be defined in a project charter at project initiation and reviewed as new members or guests join the initiative.

#### 2.1.6 Be transparent and genuine

Researchers should be transparent with youth about their objectives, goals, expectations and constraints, as well as genuine about their role and stance regarding the issues on the table, reflecting the values of youth‐adult partnerships.^17^ This includes being upfront about the timelines for the project, the number of meetings expected, the degree of input and amount of commitment the youth are being asked for, and any reasons that the researchers may not be able to apply the youth’s feedback on certain items. Similarly, researchers are encouraged to update the youth in a timely manner about any changes that occur to these factors through the course of the project. Furthermore, it is important to be transparent to youth about how their feedback is being used.^4^, ^21^ If work previously completed by or with the youth is to be changed, the researchers should connect with the youth to ensure that they are in agreement with the changes. Researchers should consider having honest check‐ins at the beginning of meetings. Furthermore, they should keep the promises they make to youth to ensure a climate of trust and transparency.

#### 2.1.7 Create youth‐friendly spaces

Engaging youth authentically requires creating a youth‐friendly space characterized by a safe, welcoming environment, where all parties’ opinions and contributions are respected and valued.^16^ This means that both the youth and the adults should feel comfortable and welcome to express themselves. Many of the tenets of youth‐friendliness in clinical mental health settings can also be applied to research environments.^22^. Specific to research environments, a youth‐friendly, welcoming space is created via clear and ongoing communication between adult researchers and the youth, polite demeanours, support and mentoring of the youth, and authentic respect for the input provided by youth.^5^ Researchers may consider whether they want the youth to refer to them by their formal title and last name (eg Dr. Smith), or whether they are comfortable being on a first‐name basis with the youth to reduce the power imbalance. The physical space is also an important consideration. It is important that the space be easily accessible and comfortable to youth^21^; as such, standard conference rooms or hospital‐based meeting spaces may not be appropriate. Researchers may consider meeting with youth in the community, for example in rooms provided by partner organizations. Ensuring that meetings are held during a time that youth can realistically attend and in an easily accessible location will help to make attendance at meetings more attainable for youth.^20^, ^21^ The physical environment can be made youth‐friendly using seating arrangements that bring youth and researchers together at the same table, on the same level, with ease of eye contact. Considering accessibility accommodations and accounting for past experiences such as trauma are also important; in some cases, the youth‐friendliness of the environment might be enhanced by having support workers available when research topics are contentious or potentially triggering.

When appropriate, research meetings can also include fidget gadgets such as pipe cleaners and stress balls, phone chargers or wireless Internet access. Having snacks available can improve the attention, productivity and friendliness of the meeting space, while also showing respect to youth for taking their time to participate. In some cases, meetings can be more creative and engaging to encourage youth participation.^21^ This can include incorporating introductions and icebreakers at the beginning of each meeting or the use of flip charts or smaller breakout discussions to facilitate conversation. When engaging with broader groups of youth, consider having a smaller number of highly engaged youth lead these consultations with support from an adult ally.^23^ When youth facilitate the consultations among their peers, the discussion becomes more youth‐focused, honest and comfortable for the young people involved. Seeing other youth in leadership positions can also increase their confidence in the project and trust that their ideas will have an impact.^21^

#### 2.1.8 Explain research concepts in jargon‐free terms

Working group discussions conducted among researchers may be driven by research jargon and concepts such as *P* values, psychometric validation or power analysis. Researchers are encouraged to reflect on their discussion points prior to meetings, identify concepts that may need to be explained and plan for the time to explain them in youth‐friendly, jargon‐free terms.^20^ If youth cannot understand the concepts being discussed, they will be unable to contribute fully to the discussion. However, while reducing the jargon they use, researchers should also avoid oversimplifying in a way that may be seen to be talking down to the youth. By listening authentically, hearing the terminology the youth use and adapting to it, and asking questions to ensure comprehension, researchers can acknowledge the youth’s experience and knowledge, while supporting optimal contributions. However, it is again important to remember that youth are not all the same. Some youth might consider the exposure to complex research terminology to be an exciting learning opportunity; this illustrates the importance of consulting with the youth to determine their goals, objectives and interests. Consider the role youth can also play in helping you make your forms and questionnaires youth‐friendly by helping with jargon, design and ease of use.

#### 2.1.9 Hold meeting pre‐briefs and debriefs

To prepare youth for research meetings and improve their ability to participate in them, pre‐brief and debrief sessions provide a valuable mentorships opportunity. Prior to a working group meeting, an adult ally on the McCain Centre research team walks the youth through the meeting agenda, explains acronyms and research concepts in youth‐friendly terms and gives the youth the opportunity to air their thoughts in a smaller forum to prepare for the meeting. Not only does this make the youth more comfortable speaking up during working group meetings, it also provides the adult ally with the opportunity to check in with the youth, understand their concerns and, when appropriate, prompt discussion on the issues raised by youth in the pre‐brief meeting. After the meeting, debriefs provide the youth with the opportunity to ask questions about the meeting content, clarify issues, reflect on their own contributions, which may help them feel more confident in future meetings.^16^

#### 2.1.10 If engaging both youth and caregivers, actively ensure the youth’s voice can still be heard

Incorporating the perspective of caregivers in research can be another valuable partnership for your project.^8^, ^24^ If you are choosing to engage both youth and caregivers in research, remember that youth and caregivers are fundamentally different groups, with different voices and needs. The dynamic of youth consultation can change dramatically if caregivers are consulted in the same room, as a power imbalance can quickly come into play. To protect the youth’s ability to express their views, consulting with youth and caregivers in separate forums is often preferable, for example, distinct youth advisory groups and family advisory groups with appropriate confidentiality guidelines to safeguard youth’s freedom to disclose. If youth and caregivers are being consulted at the same table, researchers are encouraged to ensure that the caregiver voice does not overpower that of the youth. This can be accomplished by directly soliciting youth feedback and facilitating the discussion in a manner that ensures all parties have time to express their views.

### 2.2 The “dont’s”

Despite a research team’s best intentions, youth engagement can sometimes be challenging. Researchers are encouraged to reflect on the following pitfalls that can compromise the effectiveness of their youth engagement initiatives.

#### 2.2.1 Don’t tokenize or patronize

Tokenism and patronization are key concerns among youth. When youth are not authentically engaged through open discussion, given opportunities to be heard and ensured actual involvement in the decision‐making process, this is considered tokenized engagement.^15^, ^16^ Patronization occurs when youth feel they are treated like children and their contributions are not valued. By following the “Dos” listed above, being continually aware of the risk of tokenism and being willing to change the way they think and work, researchers can ensure that the youth engagement process is mutually beneficial.

#### 2.2.2 Don’t ask for feedback, then disregard it

Authentic engagement in the decision‐making process requires not only asking youth for feedback and giving them time to express their views, but taking their feedback into account during discussions, decision‐making and project execution; this means that project timelines must allow for the time to meaningfully consider youth feedback.^14^ Youth should be made aware of how their input is influencing the project and the decisions being made, so they can appreciate the value of their contributions. When they see how their contributions have impacted a project, this can help build confidence and a sense of meaning in the work they do. When a youth’s suggestion cannot be put into action, for example when organizational policies interfere, the reasons should be made clear to the youth and alternatives should be discussed openly with them.^5^

#### 2.2.3 Don’t steer youth towards the response you want

Authentically engaging youth in research requires truly listening to their feedback and taking that feedback into account,^5^ even if it is not the feedback that the researchers were seeking or hoping to receive. If researchers have an agenda that leads them to steer youth towards the input they are hoping for, this discounts the value of the youth engagement process, leading to tokenism and the loss of any opportunity for meaningful contributions. Honest, unbiased feedback may lead a project in new and exciting directions that researchers had not initially anticipated. This illustrates some of the unique benefits that can be derived from authentically engaging youth in research projects.

#### 2.2.4 Don’t privilege one form of knowledge over another

The purpose of collaboration and consultation is to have a diversity of voices at the table. When working alongside youth, it is important to recognize that youth are experts of their own experience.^25^ Respect each of the voices brought to the table and avoid privileging one voice over another. Power imbalances can mean that decisions are driven primarily by certain forms of knowledge, for example, scientific knowledge or caregiver knowledge at the expense of youth knowledge, or the knowledge of the more privileged youth over that of the less privileged. By respecting diverse experiences and actively working to minimize the power imbalance, researchers can ensure that all forms of knowledge are leveraged to contribute to the project.

#### 2.2.5 Don’t be closed to new ideas and unwilling to adapt

Youth provide valuable expertise on the experiences and realities of youth, including the ways in which your research ideas will be received by the youth you may hope to recruit as study participants.^5^ If they disagree with a research operationalization approach, this may be a strong indicator that your approach is not youth‐friendly or feasible. By raising their concerns, they are not criticizing you as a researcher, but rather playing exactly the role they have been recruited to play. It is important to listen to their concerns and consider them without taking these concerns personally. It may be important to explain the scientific principles that underlie the researchers’ proposed approach, for example impacts on research outcomes or interactions with organizational structures, to stimulate productive dialog with the youth about the research alternatives. It may then be necessary to use critical thinking to creatively identify ways of best incorporating youth’s suggestions while maintaining scientific rigour.

## 3 CONCLUSIONS

The recommendations presented in this article aim to increase researchers’ capacity to engage youth in academic research projects, from the early planning and grant writing phases through to project completion and results dissemination. At a time when there is increasing interest in youth engagement in research,^1^, ^2^, ^3^ this article provides practical guidelines to help researchers engage youth effectively. General in nature, these recommendations can support researchers in effectively engaging youth as members of their research team in all types of academic research designs, from qualitative projects to highly complex, technical academic research initiatives. While these guidelines have not been formally validated as a model of youth engagement, they have been drawn from a wide range of literature, from youth themselves and from the progressive experience of a team that has an ongoing history of engaging youth in research.

Among the cornerstones of youth engagement is authentic respect;^16^ if researchers convey that they truly respect the lived experience of youth as experts in their own lives and in the current cultural context facing youth like them, this will set the stage for a strong youth engagement process. With respect as a backdrop, researchers can optimize the opportunities provided through youth engagement by focusing on clear communication and transparency, while adapting their processes to set the stage for success. Researchers are encouraged to remain mindful of the risk of tokenizing the youth they mean to engage, which would compromise the engagement process.^15^

Youth can and do provide valuable input into academic research projects, making the research conducted about them more relevant. Researchers are encouraged to reflect on the value that youth engagement can bring to their social research practices. By engaging youth authentically in the research conducted about them, researchers can increase the applicability, feasibility, youth‐friendliness and ecological validity^3^, ^4^ of their work and ultimately improve the value and impact of the results their research produces. By encouraging the development of collaborative, youth‐engaged research agendas, these practical recommendations aim to vitalize youth engagement in research.

## CONFLICT OF INTEREST

The authors have no conflict of interests.
